# Propagating annotations of molecular networks using *in silico* fragmentation

**DOI:** 10.1371/journal.pcbi.1006089

**Published:** 2018-04-18

**Authors:** Ricardo R. da Silva, Mingxun Wang, Louis-Félix Nothias, Justin J. J. van der Hooft, Andrés Mauricio Caraballo-Rodríguez, Evan Fox, Marcy J. Balunas, Jonathan L. Klassen, Norberto Peporine Lopes, Pieter C. Dorrestein

**Affiliations:** 1 Collaborative Mass Spectrometry Innovation Center, Skaggs School of Pharmacy and Pharmaceutical Sciences, University of California, San Diego, La Jolla, CA, United States of America; 2 NPPNS, Department of Physic and Chemistry, School of Pharmaceutical Sciences of Ribeirão Preto, University of São Paulo, Ribeirão Preto, SP, Brazil; 3 Bioinformatics Group, Department of Plant Sciences, Wageningen University, Wageningen, The Netherlands; 4 Department of Molecular and Cell Biology, University of Connecticut, Storrs, CT, United States of America; 5 Division of Medicinal Chemistry, Department of Pharmaceutical Sciences, University of Connecticut, Storrs, CT, United States of America; Icahn School of Medicine at Mount Sinai, UNITED STATES

## Abstract

The annotation of small molecules is one of the most challenging and important steps in untargeted mass spectrometry analysis, as most of our biological interpretations rely on structural annotations. Molecular networking has emerged as a structured way to organize and mine data from untargeted tandem mass spectrometry (MS/MS) experiments and has been widely applied to propagate annotations. However, propagation is done through manual inspection of MS/MS spectra connected in the spectral networks and is only possible when a reference library spectrum is available. One of the alternative approaches used to annotate an unknown fragmentation mass spectrum is through the use of *in silico* predictions. One of the challenges of *in silico* annotation is the uncertainty around the correct structure among the predicted candidate lists. Here we show how molecular networking can be used to improve the accuracy of *in silico* predictions through propagation of structural annotations, even when there is no match to a MS/MS spectrum in spectral libraries. This is accomplished through creating a network consensus of re-ranked structural candidates using the molecular network topology and structural similarity to improve *in silico* annotations. The Network Annotation Propagation (NAP) tool is accessible through the GNPS web-platform https://gnps.ucsd.edu/ProteoSAFe/static/gnps-theoretical.jsp.

## Author summary

For genome analysis it is commonly accepted that one can hypothesize the function of genes based on sequence similarity, using annotated reference sequences. Once a homology hypothesis has been made based on reference annotations, it allows one to build hypothesis in terms of function and ultimately understand the underlying biology. In contrast, mass spectrometry (MS) can detect many molecules, as ions, yet we often cannot link a MS signal to a molecule. The reference libraries to annotate fragmented molecular data only cover a small portion of the known molecular space. The use of computational (*in silico*) fragmentation predictions from structural libraries offers a promising alternative. One of the weaknesses of the molecular annotation using such *in silico* approaches is that they currently annotate the molecules individually. However, molecular relationships, based on spectral similarity, can be used to enhance the structural hypothesis inferred from the annotation of molecules detected by mass spectrometry. We introduce an online tool called “Network Annotation Propagation” that uses a combination of molecular networks, based on spectral similarity, from which we infer molecular similarity, together with *in silico* fragmentation, to enable the scientific community to strengthen their MS annotations.

## Introduction

One way to gain insight into the molecules of a biological sample is through mass spectrometry. Mass spectrometers are incredibly sensitive equipment capable, under specific conditions, of measuring attograms (10^-18^g) of molecules in a sample [1]. In a targeted mass spectrometry analysis, such as airport security scans, for example, only molecular signatures of predetermined compounds (e. g. explosive components) are searched. In an untargeted mass spectrometry experiment, we do not set the mass spectrometer to weigh specific molecules only, instead, we have the potential to observe hundreds to thousands of ions from a single sample; but most experiments report only on one or a few dozen molecules and often within the limit of known pathways described in textbooks [2]. However, such pathways represent only a fraction of molecules that are detected.

Untargeted mass spectrometry is usually performed as follows: liquid chromatography based infusion is led into the instrument, then the ions are isolated inside the mass spectrometer, accelerated in a chamber filled with helium gas, for example, which results in thermal activation due to the collisions with the gas. When the ion is sufficiently activated, some of the molecule’s bonds break and the resulting fragments can be observed. Untargeted mass spectrometry of laboratory cultured organisms, or even cells is already very complex, and this complexity increases in real world samples from plants, animals (including humans) and environments, as the molecules can come from the host cells, diet, their microbiome as well as any other environmental exposure. The way we convert the fragmentation spectrum of fragmented molecules, also referred to as an MS/MS, to identifications is through matching the observed MS/MS spectra to spectra from reference MS/MS libraries, termed spectral library search. The goal of the spectral library search is to find the best MS/MS match between an unknown spectrum and a known spectrum of a previously characterized molecule in the library. On average, through matching fragmented spectra with reference libraries, we can annotate 2% (average of spectral library matching in all GNPS datasets) of the data [3], although for well studied biological matrices, such as *Escherichia coli*, human cell lines, plasma or urine this may be as high as 10% [4].

**Fig 1:**
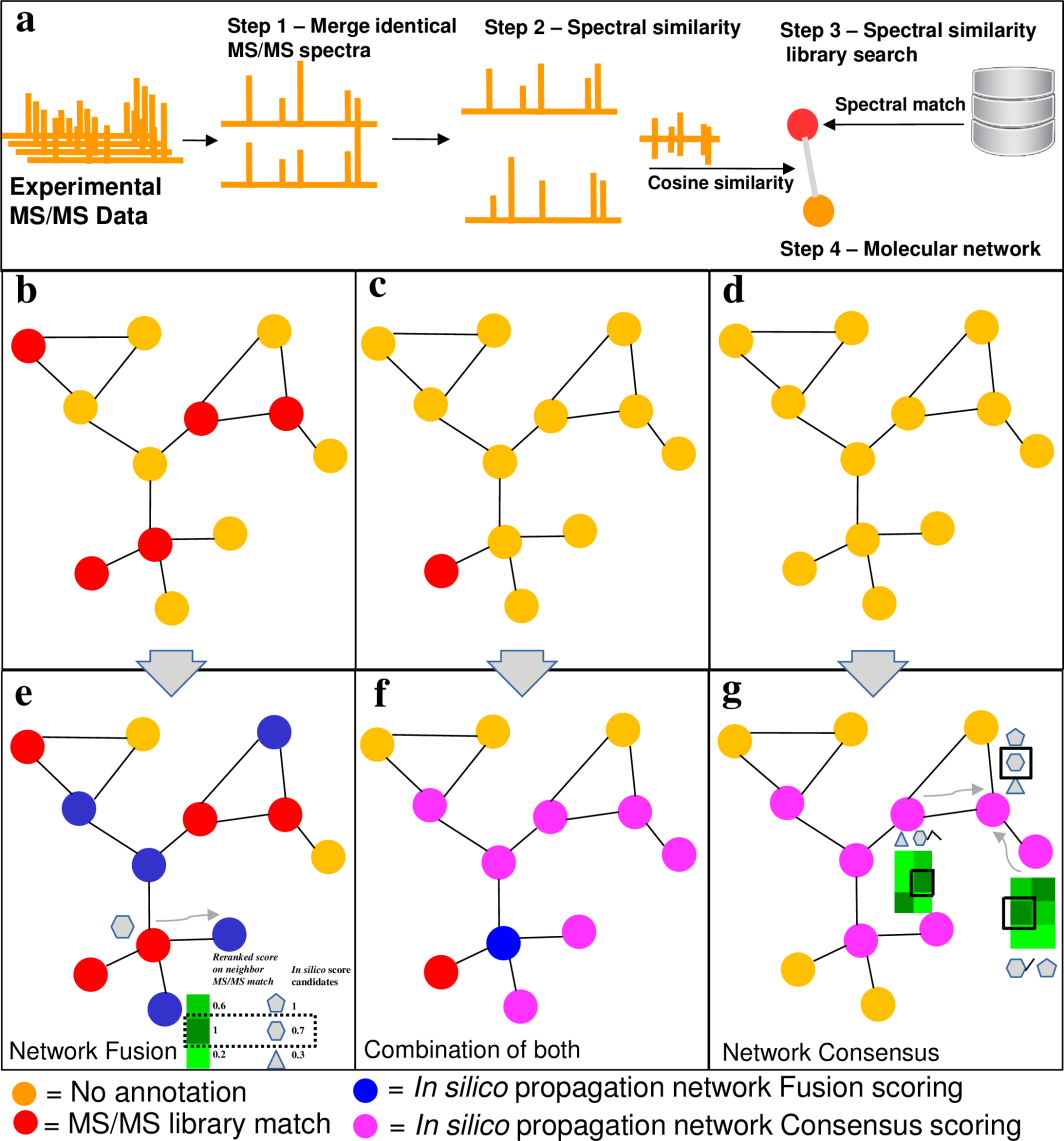
Representative scenarios of molecular networks obtained in an untargeted MS/MS experiment and possibilities for propagation. a) Introduction of molecular networking and library matching. b, c and d represent varying degree of spectral annotation in the network. e, f and g illustrate how the propagation of annotations can be used for each respective scenario (represented in the top panel). e) The *Fusion* scoring—The spectral library hit nodes (red) are employed to recalculate the score of candidate structures (grey shapes associated to nodes) for nodes having structure candidates from *in silico* fragmentation search (blue), based on their structural similarity (Represented by the green heatmaps, where darker green indicates a higher degree of similarity). f) and g) The *Consensus* scoring—a *Consensus* scoring can be used, based on the joint similarity of neighbors (pink nodes) for spectral library hits and i*n silico* annotations (f), or *in silico* annotation only, when no library match is present (g).

To better understand this complexity, we must be able to increase our annotation rates of spectra across all projects in the public domain. Using spectral alignment to create molecular networks [5], molecules can be grouped into molecular families (inferred structural analogs) and annotations from known compounds can be propagated to connected spectra (neighbors) in the network (Fig 1A). While molecular networking can double or even triple the annotation rates [5], it requires a tedious process of manual inspection [6]. Reference libraries are commonly generated from commercially available standards, which resulted in very biased molecular libraries in the public domain as well as commercial libraries. While we have begun to capture annotations of spectra by the community from unpurified material, using the expertise of public data depositors [5], one of the most promising ways to improve annotations is through *in silico* matching to structural databases covering a broader range of the chemical space. Computational tools are able to capture and infer structural information from mass spectrometry data at a scale that dwarfs manual inspection rates. Although a variety of methods are used for *in silico* fragmentation matching [7], usually multiple candidate structures match a given query spectrum. Several *in silico* fragmentation methods have proposed different criteria to rank the most likely candidates, however, currently the correct structure is in average ranked among the first tens of candidates [8]. This means that an end user still has to wade through the top k matches and visually inspect the predictions, which is one of the reasons why such approaches are slowly adopted by experimentalists. In the last ten years the development of *in silico* fragmentation methods is experiencing a great improvement, in part because experimental data and reference libraries have become available in the public domain, providing training data for increasingly sophisticated algorithms to be developed [9–12]. The development of the next generation *in silico* fragmentation annotation methods hold the promise to increase the number of annotations and make this process much more efficient [13], however we often have multiple candidate structures predicted for each fragmentation spectrum and an end user does not have a good way to prioritize the candidate matches. Although there are other approaches to propagate annotations in a mass spectrometry experiment [14–18], since the introduction of molecular networking in 2012 by our labs, we and others have demonstrated that the concept of propagation through spectral alignments works extremely well [19–25].

We therefore set out to explore if a large portion of the “dark matter” of metabolomics [3] experiments can be uncovered by combining molecular networking with existing *in silico* methods to improve the annotation rates and quality through automated propagation. An early hint of the utility of this approach was recently demonstrated. The Wolfender lab combined an *in silico* library using the *in silico* fragmentation method CFM-ID [9], and molecular networking results obtained through our community analysis platform Global Natural Product Social (GNPS) infrastructure, followed by subsequent manual inspection of the results. The Wolfender lab demonstrated that the combination of spectral networks and *in silico* fragmentation was an effective strategy for dereplication, a term used for identification of known molecules [26]. While this work explores the advantage of the combination of two annotation approaches, the *in silico* prediction method did not yet take advantage of the network topology. The topology should be taken in account, once, under the assumption that neighbor nodes in the spectral networks are structurally related, the *in silico* annotation of neighbor nodes should result in structurally related candidates. Here we show that the ranking of *in silico* annotations using the *in silico* prediction tool MetFrag [12, 27], can be improved using the topology of the molecular network through building consensus among the candidate structures from neighbors in the network. We further show this is effective even without any spectral library match. The approach is called Network Annotation Propagation (NAP). There are two scoring approaches utilized by NAP to re-rank candidates. When there is a spectral library match within a molecular family of the molecular network (connected component of a graph), NAP utilizes the MetFrag *in silico* prediction with the MetFusion [28] score to re-rank candidates (which we term *Fusion* scoring) (Fig 1B and 1E). The *in silico* fragmentation tool MetFusion combines the output of spectral library search and *in silico* fragmentation predictions by MetFrag to improve candidate structure ranking, by taking into account the structural similarity of all *in silico* candidate structures to spectral library candidate structures. In NAP, the MetFusion spectral library matches are replaced by the annotations of all direct neighbors in the network. When there are no or very few spectral library matches (Fig 1C and 1D), a network consensus scoring will obtain the structural similarity from the candidate structures instead, exploiting the structural similarity of the *in silico* candidates (which we term *Consensus* scoring). This means that it is now possible to propagate annotations even when there are no spectral matches to reference MS/MS data.

After ranking improvements by considering neighboring nodes in the network, we show, using a spectral network of known spectra as benchmark data, that unsupervised cluster detection of candidate structures ranked using the network *Consensus* and *Fusion* scoring can find up to 81% of the correct compound substructures present in the first ranked candidate within a candidate structure list when reference libraries are considered while up to 63% correct compound substructures present in the first ranked candidate are found when no reference libraries matches are available within the molecular network. Additionally, because it takes significant computational resources for both data storage and to compute the propagations, NAP has been implemented as a ProteoSAFe workflow for High Performance Computing (HPC) onto the GNPS analysis infrastructure at the UCSD Center for Computational Mass Spectrometry.

## Results

### Construction of network Fusion and network Consensus in NAP

The resulting molecular clusters of molecular networking can be categorized into three different scenarios. First, clusters where we have a large number of matches to reference MS/MS spectra (Fig 1B), secondly, clusters with one or very few spectral matches by connected component (Fig 1C), and finally clusters with no spectral matches (Fig 1D). This range of scenarios requires different solutions. NAP was designed to handle all three scenarios. The starting point for NAP is the construction of a molecular network from data containing MS/MS spectra on the GNPS web-platform. Nodes in the network correspond to clusters of similar fragmentation spectra and edges represent spectral similarity between any two given nodes [5] (Fig 1A). Molecular networks allow the generation of hypothesis regarding the structural relationship of compounds connected in the network through annotation propagation. In parallel to molecular network construction, each node (a consensus spectrum) is subjected to spectral library search and *in silico* fragmentation search using MetFrag (Fig 1E–1G). The re-ranking of *in silico* candidate structures is calculated by the weighted contribution of MetFrag's score with a “spectral summary” (see Methods section), as previously described in MetFusion [28] and therefore we refer to this approach as network *Fusion*. We extended the *Fusion* principle to regions of the network where there are no library hits, where the *in silico* candidate structures of a given node can be re-ranked, by finding which candidate structure for this node maximizes the structural similarity to its neighbor node's candidate structures (Fig 1G). The *Fusion* scoring has a strict requirement that a library match has to be available as a direct neighbor (Fig 1B). However, in the case of sparse library matches in a molecular network or none at all (Fig 1C and 1D), we can apply network *Consensus* scoring (Fig 1G). The consensus scoring is built from the *n-first* neighbor candidates, where *n-first* is a user selected parameter. It is also possible to combine network *Fusion* scoring with network *Consensus* scoring through NAP (Fig 1F). In that scenario network *Consensus* scoring is calculated after network *Fusion* scoring and uses its re-ranked scores, instead of MetFrag’s, to propagate the spectral annotations to more distant neighboring nodes.

### Benchmarking NAP with a standard library

To benchmark NAP, we have created a molecular network with a subset of 5,467 MS/MS [M+H]^+^ spectra from NIST17 library that are structurally unique and have spectral similarity (cosine score > = 0.6) to at least one spectrum in the subset (Fig A in S1 Text). A further subset (1,734 spectra), consisting of a network with only edges having a cosine score < 0.7 was selected in order to ensure that the annotation propagation will not be biased by structural identity [28], and the re-ranking can be performed with varying degree of structural similarity to neighbor nodes. This validation is important for analogs that share a substructure that is captured by spectral similarity, and helps to show that the propagation can be useful in that scenario (Figs B and C in S1 Text). NAP is built on top of *in silico* fragmentation performed with MetFrag, which searches for biologically relevant small molecule in a structure database including GNPS, HMDB (Human Metabolome Database) [29], SUPER NATURAL II [30], ChEBI (Chemical Entities of Biological Interest) [31] and DNP (Dictionary of Natural Products). In total this represents 367,204 unique small molecules (based on the first block of InChIKeys).

**Fig 2:**
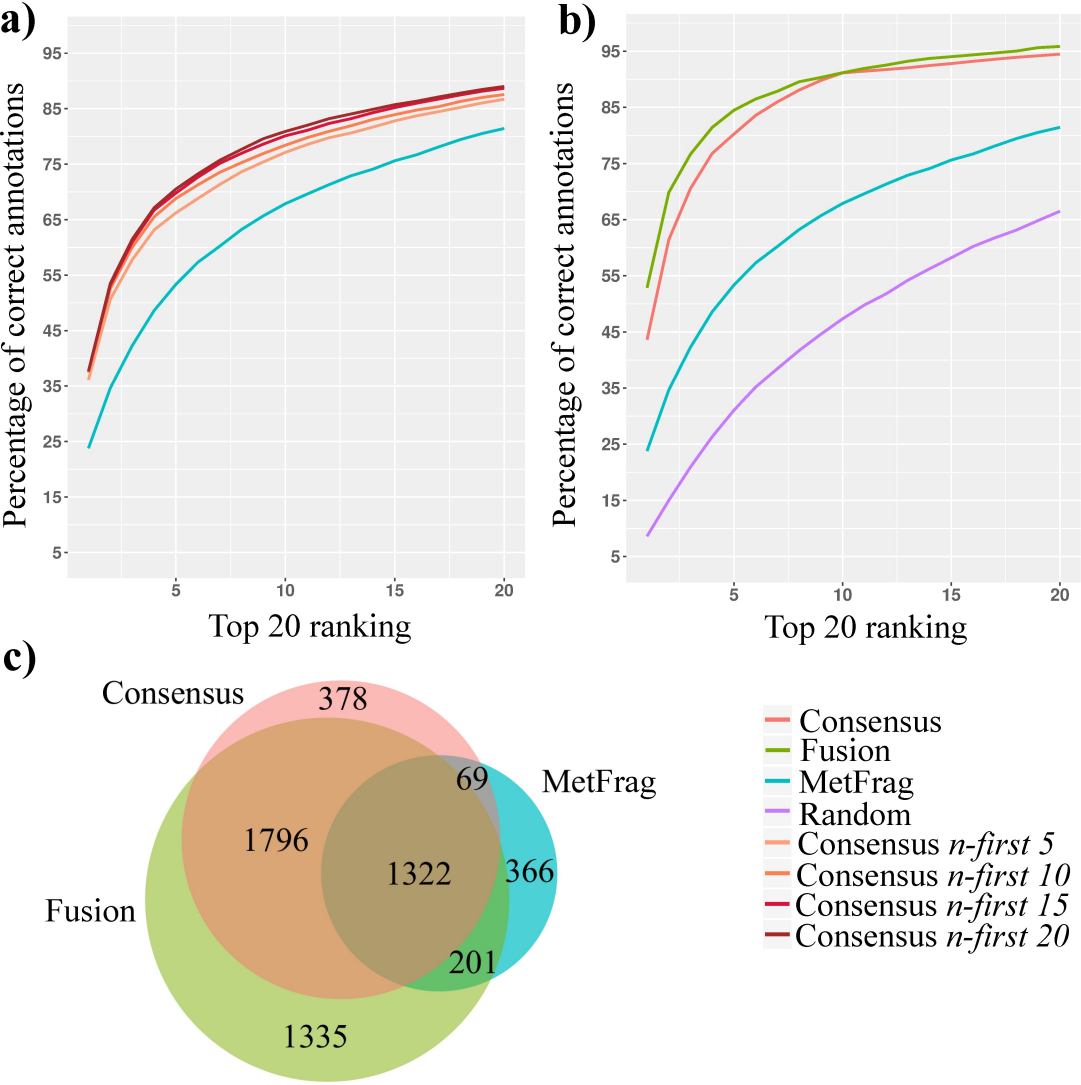
NAP re-ranking assessment using the 5,467 NIST17 [M+H]^+^ benchmark data set that have known nearest neighbors in the molecular network. a) The impact of setting the *n-first* parameter on percentage of correct annotations for network *Consensus* scoring. Where *n-first* indicates the *n* number of top ranked candidate structures (from 5 to 20) considered from the neighbor nodes during the *Consensus* scoring. b) Percentage of correct annotations ranking for each method. c) Number of spectra with improved ranking of correct annotations, that is, for which *Fusion* or *Consensus* scoring ranked the correct structure better than MetFrag.

First, we created ranked candidate lists for each spectrum (node in the network) individually with MetFrag, which were used as the base ranking for propagation. Departing from candidate ranking at single spectrum level we wanted to assess the impact of network *Consensus* scoring on the results. In order to assess the influence of the *n first* parameter on the network *Consensus* results, we applied the *Consensus* ranking method and varied the *n-first* parameter from 1 to 20 (Fig 2A and Fig D in S1 Text). Because network *Consensus* does not consider spectral library matches, the resulting ranking is based on the re-ranking of structures obtained with MetFrag based on the propagation of all direct neighbors’ structural similarity. If neighbor nodes deriving from highly similar spectra not grouped in the networking process (Fig 1A—Step 1) are present, and structurally similar candidates are present in the candidate list, *Consensus* scoring can help to obtain structurally related candidates (Fig E in S1 Text). By adjusting the *n-first* parameter, it is possible to observe the relationship between the number of neighbor structures considered with the ranking position of the correct structure of the node being re-ranked (S1 Data).

The ranking of known molecular structure with MetFrag alone at single spectrum level had a mean ranking position of 14.7 with a median of 5 (Fig 2B). If we only consider the top 5 ranked candidates of all direct neighbors with *Consensus* (*n-first 5*), we observe an average ranking improved to 10.9 and median to 2. Further improvement was observed increasing the *n-first* parameter to *10*, improving mean to 10.2 and median to 2. Similarly, for *n-first* parameters *15* and *20* we observed means of 9.8, 9.6 and median of 2 and 2, respectively (Fig 2A, Fig D in S1 and S2 Data). Thus, the *Consensus* scoring was able to annotate the correct structure in median on the top two candidates searching a reasonable large structure database derived from biological sources (367,204 molecules). Looking at Fig 2A one could conclude that a higher the *n-first* parameter always improves results, which is not necessarily the case, for example, inspecting unique candidates ranked in the first positions by different parameter numbers shows that *n-first 1* had more unique correct compounds better ranked than *n-first 5* to *20* (Fig F in S1 Text). The effect of the *n-first* parameter depends on the average number of candidates obtained as well as the number of connections the nodes have, as each node takes information from all directly connected nodes. Although we offer a default value of 10 *n-first*, that parameter has to be adjusted for each study, and ideally, manually curated.

Next we set out to assess the other approaches in NAP, the impact of network *Fusion* scoring, and from *Consensus* scoring (using as base ranking the *Fusion* scores) with *n-first* parameter fixed to 10. The network *Fusion* score had 29.0% increase in the first position ranking and overall 18.4% increase on rankings better than MetFrag alone for correct annotation among the top twenty candidates (Fig 2B and S3 Data and S4 Data). If *Consensus* scoring is being applied it means one or more neighbors possess *in silico* fragmentation candidates, and those are used to re-rank the candidate list of the node being processed, under the assumption that nodes connected by spectral similarity should possess structurally related first ranked candidates. For instances where the direct neighbor was previously re-ranked by *Fusion* scoring, the *Consensus* scoring can take advantage of this previous ranking, as the correct structure is more likely to be ranked among the top *n-first* candidates (Fig 1F). The network *Consensus* scoring had a 19.8% increase in the first position ranking and overall 13.0% increase on rankings better than MetFrag alone for correct annotation among the top 20 candidates, when propagating is performed after *Fusion* scoring (Fig 2B and S3 Data and S4 Data).

The subset with edges of cosine score < 0.7 had very similar results to those described above for the complete network (S5 Data and Fig G in S1 Text), showing that the propagation is efficient under varying degree of spectral similarity. Overall, the average ranking of the correct annotation improved from 14.7 for MetFrag alone (and rank 23 for random assignment) to 4.7 for network *Fusion* and 6.3 for network *Consensus* (using previous *Fusion* ranking). The median improvement ranged from 5 for MetFrag alone (and rank 12 for random assignment) to 1 for network *Fusion* and 2 for network *Consensus* (Fig 2A and S4 Data and S5 Data). Both network *Fusion* and network *Consensus* scoring also have a larger number of unique (best ranking observed only for one approach—*Fusion*, *Consensus* or MetFrag) correct rankings when compared to MetFrag (Fig 2C). Interestingly, although 32.8% of improved annotations were found by network *Fusion* and network *Consensus* overlap, network *Consensus* found 6.9% annotations with better ranking than network *Fusion*, while network *Fusion* had 24.4% better annotations (Fig 2C). Those results suggest that, even in a network with nodes having spectral library matches, the use of neighbor candidate structures can provide complementary information for ranking.

### Clustering candidate structures

**Fig 3:**
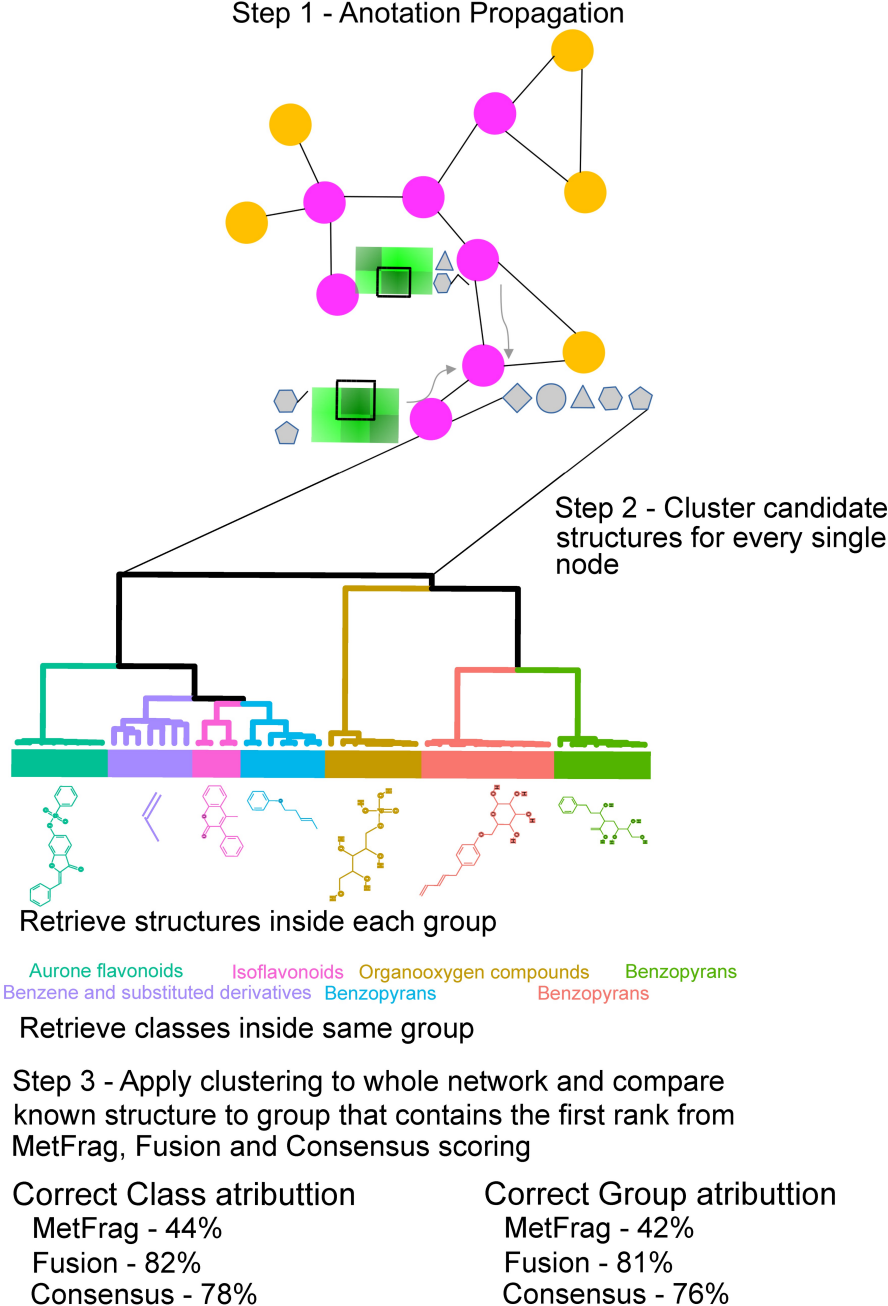
Schematic representation of the molecular structure candidates clustering by structural similarity and dynamic cluster assignment from the 5467 NIST17 [M+H]^+^ unique compound spectra with ClassyFire chemical taxonomy. The structurally related group of candidate structures, detected by unsupervised clustering, containing the first candidates ranked by network *Fusion* and network *Consensus* often contains the maximum common substructure shared between *in silico* candidates (for candidates inside the group defined by clustering) and the known structure in the validation dataset (numbers shown in the bottom). The structurally related groups are highlighted by colors, inside each group we also show class assignments. The classes/structures that were predicted by unsupervised clustering were compared with the known compound.

To further validate the approach we tested whether NAP annotations were correct at substructure level. In order to group candidate structures based on their structural similarity and dynamically assign groups inside candidate lists we used the Dynamic Tree Cut method [32] for dynamic branch cutting and unsupervised group detection on the result of hierarchical clustering (Fig 3). Grouping the structurally related candidates revealed that 84% of the structures assigned as first candidate by network *Fusion* scoring were contained in the same ClassyFire [33] chemical *Class* taxonomic classification of the known true structure (Correct Class), compared to 44% for MetFrag only (Fig 3, S6 Data). The ClassyFire classification provides a hierarchical classification that, similarly to unsupervised grouping, is based on structural similarity, however, specific features of the compound classes can be captured by nested classifications, from *Super Class* to *Class*, for example (See Methods section for user manual link). For network Consensus 78% were contained in the correct *Class* (66% considering *Consensus* without *Fusion*). When the structural similarity group is considered with multiple ClassyFire chemical classes, 84% of the structures assigned as first candidate with network *Fusion* and 76% for network *Consensus* scoring (67% considering *Consensus* without *Fusion*) were contained in the same structure similarity group of the known true structure (Correct substructure), compared to 42% for MetFrag only (Fig 3) suggesting that *in silico* methods can benefit from chemical classification as the best ranked structure is more likely to share a substructure with correct structure and belong to the same chemical class.

### Single spectrum searches

We tested the NAP on its ability to annotate the challenge spectra from the Critical Assessment of Small Molecule Identification (CASMI) contest 2016 (http://www.casmi-contest.org/2016/index.shtml). The CASMI contest aims at benchmarking computational tools in untargeted mass spectrometry. The CASMI 2016 consisted of 146 spectra in positive ion mode, and 81 spectra in negative ion mode. The CASMI spectra were combined with all public spectral MS/MS libraries (after removal of CASMI library) to form one molecular network (one for each acquisition mode). The public MS/MS libraries were added as NAP needs a spectral network as input and will only be able to propagate an annotation if two or more similar spectra are connected nodes in the network. After molecular networking, only the CASMI spectra and their connected nodes were retained. The filtering resulted in 136 positive and 50 negative spectra with at least one putative analog connected in the spectral library network. This means not all data from CASMI 2016 had a related structure in the public libraries, especially for negative mode that has fewer MS/MS references in the public domain. The resulting network contained 884 and 175 nodes for positive and negative modes respectively (Fig H in S1 Text). In the network, 33 positive and 4 negative spectra from CASMI were connected to at least one other challenge spectrum. The networks were composed of 107 and 44 molecular families (or connected components) for positive and negative nodes, respectively (Fig H in S1 Text). NAP was able to annotate 129 structures (6 structures had spectral analogs with names only and no structural information annotated with InChI or SMILES), 113 correct structures were ranked in first position, 10 on second, 4 in third, 1 in fourth and 1 in tenth place. Overall NAP, using reference library annotations (*Fusion* ranking), had 54 better rankings than MetFrag alone (S7 Data). When reference libraries were not directly used (*Consensus* ranking) NAP was able to annotate 130 structures (5 structures had spectral analogs with no structural information annotated), 70 correct structures in first position, 19 on second, 6 in third, 10 in fourth and 25 from fifth to tenth place. Overall network *Consensus* had 36 better rankings than MetFrag alone (S7 Data). For negative mode, NAP using *Fusion* scoring had one structure not annotated, 44 in first place, 4 in second and 1 in fifth place, overall 19 better rankings than MetFrag alone (S8 Data). For *Consensus* scoring in negative mode, we found one structure not annotated, 31 in first place, 7 in second and 11 from third to tenth place, overall 8 better rankings than MetFrag alone (S8 Data). Remarkably, even though we considered only 186 out of 227 challenge spectra, the strategy of network annotation propagation using MetFrag annotations had a comparable performance to the best performing employed in category 3 of the CASMI 2016 [8], highlighting that molecular networking and re-ranking based on structural information can complement such existing *in silico* fragmentation methods. The cumulated numbers (indicated below between parentheses) of correct annotation with the top rank in positive and negative ionization modes were, for NAP using network *Fusion* (157), for the network *Consensus* scoring (using previous *Fusion* ranking) (101), for MS-Finder [34] (159) and CFM-ID [157]. Note that NAP strategy with the spectral library network made from public spectral libraries can be computationally intensive, especially when large number of spectra are used. For that reason, we implemented NAP interface in the GNPS interface that uses high performance computing. Step by step instructions on how to use NAP is provided in the supporting information (Supplementary Tool Manual) and online (https://gnps.ucsd.edu/ProteoSAFe/static/gnps-theoretical.jsp).

### Assessing the utility of NAP with metabolomics data sets

Above NAP has only been tested with known reference standards but not against typical data sets that are encountered in untargeted metabolomics experiments. We now set out to test NAP against previously published public fecal and plant metabolomics data sets from GNPS (MassIVE IDs: MSV000081120 and MSV000080502) [35–37] and a new fungal data set (MSV000081671). We chose these sets because authors of these projects had annotated them extensively. Fecal samples, analyzed with a Thermo Q Exactive instrument, represent complex mixtures full of small molecules from various backgrounds including endogenous compounds like amino acids, sugars, drugs and other xenobiotics, and food derived compounds. When analyzing molecular networks derived of such sample types, although the data sets have been inspected in great detail, there are still many molecular families [38, 39] that currently have few library matches or none at all.

**Fig 4:**
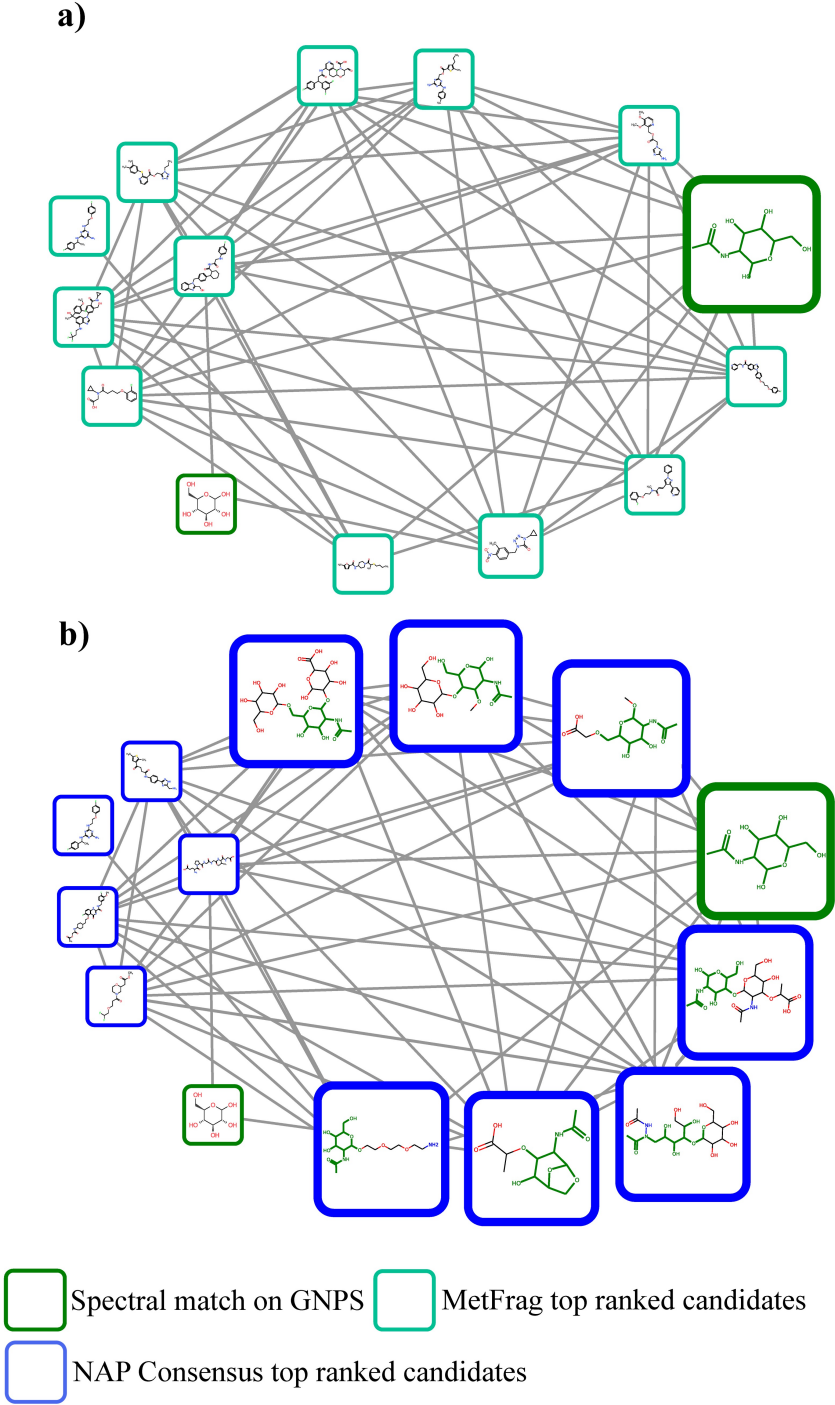
N-acetyl-sugar metabolite family: Annotations of library match to N-acetylgalactosamine propagates through network. N-acetylglucosamine containing top-ranked candidates are represented in larger boxes. **a)** Result from MetFrag **b)** Result from network *Consensus*. Highlighted in green is the maximum common substructure (MCSS) of each node to the reference library (green border node) for the seven N-acetylglucosamine related molecules.

We therefore set out to test NAP using the structural databases of natural products collection described above as well as PubChem. One molecular family that consisted of 14 nodes included two reference library matches: one to N-acetylgalactosamine and one to glucose from the GNPS-EMBL-MCF spectral library (Fig 4). Both library matches initially result in level 3 annotation according to the guidelines forwarded by the metabolomics society in 2007 [40]. Manual inspection revealed that the node with parent mass 222.110 is indeed consistent with N-acetylgalactosamine (Fig I in S1 Text) but N-acetylglucosamine is a likely candidate as well because they exhibit similar fragment losses (Fig I in S1 Text) consistent with the level 3 annotation. A comparison with standards would be required to achieve level 1 annotation [40]. Manual inspection of the data could not support the match to glucose (Fig J in S1 Text) and we would consider this to be a false annotation as i) with the search parameters used we do not have a match with FDR of 1% [41] and ii) glucosamine also results in mass fragments typical for sugars. In total, five propagated nodes presented N-acetylglucosamine containing structures and two additional structures had sugar and acetate structural features, albeit one structure had two acetates and the sugars are not in a cyclic configuration and are of different size.

Upon matching the MS/MS spectrum of the node with precursor *m/z* of 294.118, each of the candidate hits matched by MzCloud (www.mzcloud.org) contained N-acetylglucosamine (or N-acetylgalactosamine) (Fig 4 and Fig L in S1 Text). Further inspection of the fragmentation of N-acetylglucosamine revealed that the specific N-acetyl containing fragments with *m/z* 96.0444 and 84.0444 (both [M+H]^+^) are present across all members of the subnetwork. Some nodes did not return a N-acetylglucosamine containing structure within the top-10 candidates, it is likely that for those precursor masses no N-acetylglucosamine containing candidates are present in the reference structure library. Thus, the key take away from this NAP result is that this fecal data set contains a putative N-acetylated sugar family of molecule. Finally, it is worth noting that none of the top candidates found by MetFrag alone contained N-acetylglucosamine substructures, indicating how the propagation of a library match within a molecular network positively contributes to candidate ranking (Fig 4).

**Fig 5:**
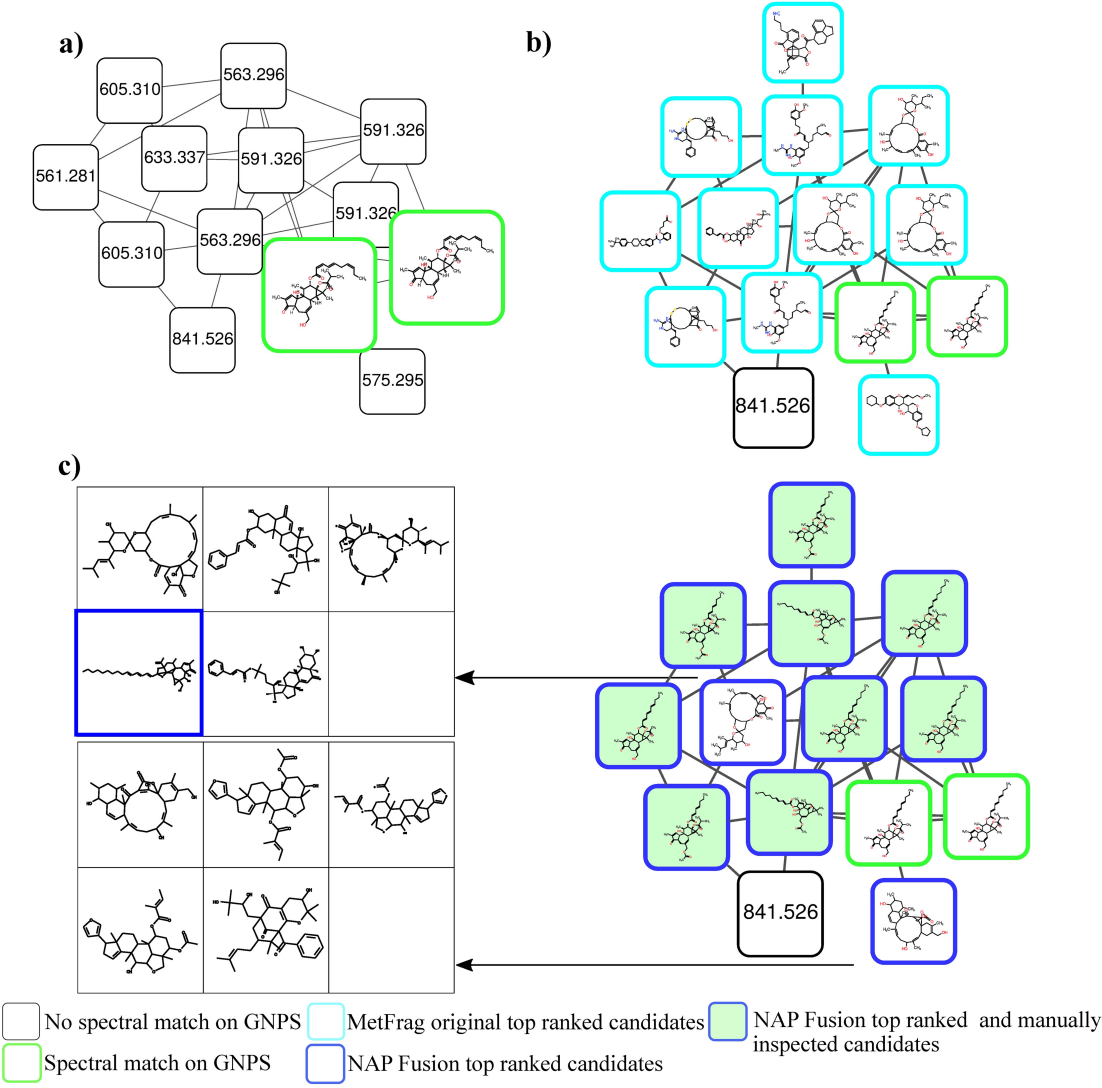
Network annotation of the *E*. *dendroides* plant extracts with NAP and visualized in Cytoscape with ChemViz2 plug-in. a) Result of the spectral library annotation using public spectral libraries available on GNPS. b) MetFrag annotation with top scoring molecules from bio-database. c) NAP annotation with top scoring matches using NAP network *Fusion* scoring, showing candidate lists associated to two nodes.

To further highlight the potential of NAP to aid structural annotation, we also processed a previously described data set [36] from extracts of the plant *Euphorbia dendroides*. In Fig 5, we illustrated how the network propagation from known molecular structures with available MS/MS spectra can improve the candidate ranking of the neighboring nodes. The reference MS/MS spectra are obtained from 23 compounds isolated from that extract and subsequently identified by NMR [36, 37], some of which were already available in the GNPS spectral library. The GNPS network revealed that 18 previously identified molecules in this dataset were annotated. For one molecular family we observed two spectral library matches, both belonging to the phorboid diterpene esters, while none of the other nodes in the network were annotated with spectral library search (Fig 5A). Among the candidates proposed by MetFrag (Fig 5B) with the structure bio-database described above, none of the top ranked were phorboids diterpene esters, while 11 out of 14 nodes were annotated as phorboid derivatives by network *Fusion* based re-ranking (Fig 5C). When *Consensus scoring* was used without taking in account the library matches, 8 out of 14 nodes were correctly annotated (Fig M in S1 Text).

**Fig 6:**
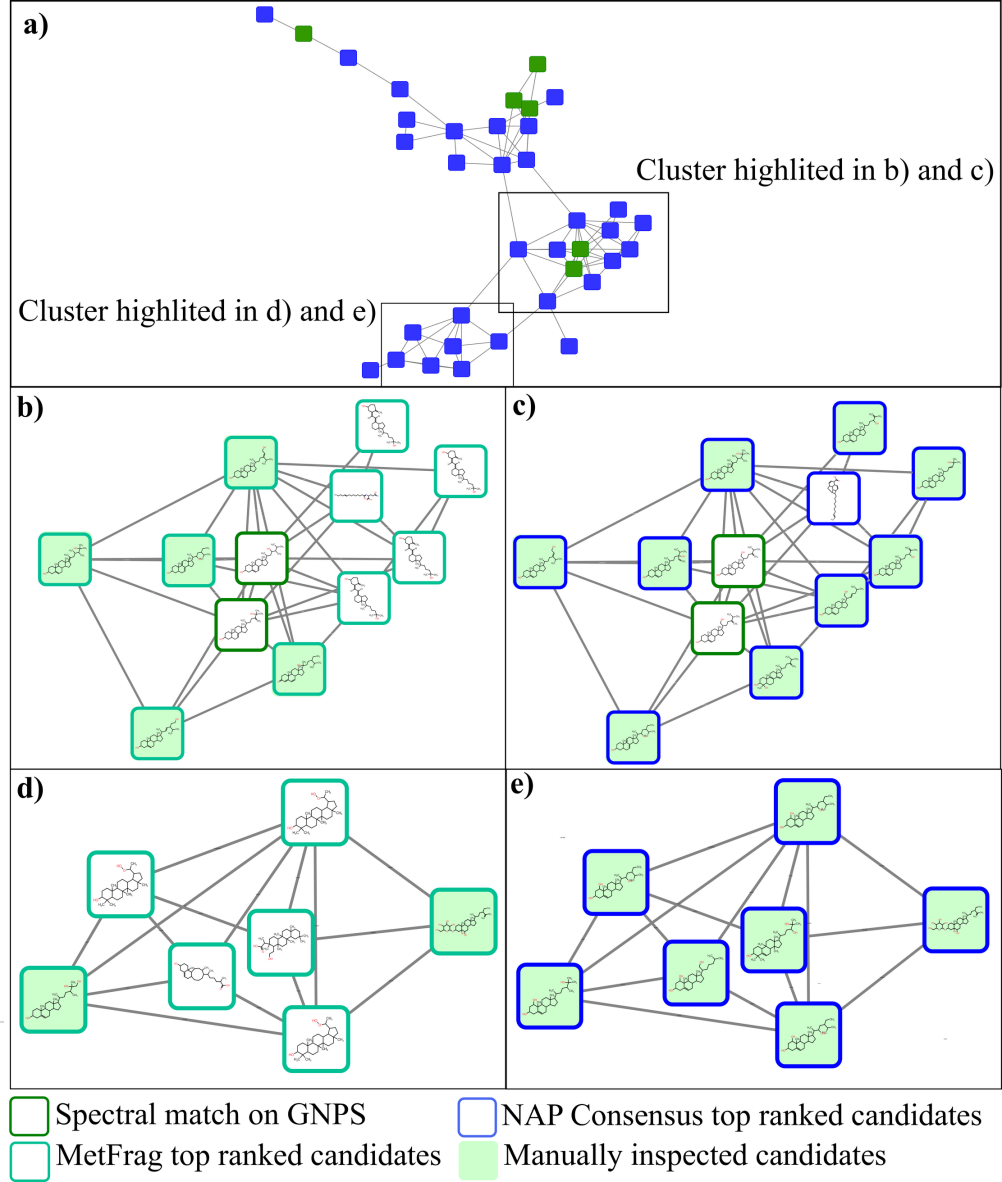
Network annotation of the *T*. *septentrionalis* fungus gardens extracts with NAP and visualized in Cytoscape with ChemViz2 plug-in. a) Group of nodes in which the annotation can be directly propagated from spectral library matches (top) and other in which the inspection of neighbor candidate structures can improve the annotation (bottom). Blue background nodes represent the presence of candidate structures from *in silico* annotation and green background represents candidates from spectral library annotation. b) MetFrag top ranked candidates. c) NAP consensus top ranked candidates. d) MetFrag top ranked candidates. e) NAP consensus top ranked candidates.

The third data set was obtained from fungus gardens raised by the ant *Trachymyrmex septentrionalis*. A 37-nodes molecular family was selected as illustrative example of the network annotation propagation workflow for this data set (Fig 6). GNPS library matches suggested oxygenated steroid derivatives present in this cluster. Manual verification allowed confirmation of the annotation for ergosterol peroxide (*m/z* 429.336) with a parent accurate mass (error 0.4 ppm), while an ergosterol derivative was suggested for the *m/z* 415.357 node (Fig 6). We used NAP to search the DNP database for related structures. *Consensus* scoring provided additional structural annotation for 32 nodes related to ergosterol peroxides, while MetFrag provided top ranked annotations for only 18 spectra (S9 Data). Fig 6 shows example annotations for a cluster of nodes that reproduce the theoretical scenario illustrated in Fig 1F, where spectral library annotations can be propagated to direct neighbor nodes, that aid in the propagation to more distant nodes with *Consensus* scoring.

## Discussion

One of the exciting developments in the annotation of highly complex samples derived from an organism or environment, is to take advantage of the relatedness of molecules co-occurring in the samples, which are often substrates and products of biochemical transformations. The benefits of using chemical relatedness to improve LC-MS/MS-based annotation using expected biotransformations was demonstrated by different approaches in the metabolomics field [17, 42–48]. Herein, we show that annotation propagation using molecular networking improves the annotations of neighboring nodes, harnessing the expected relationships of compounds detected in the sample. The process of propagating is often guided by the mass difference between the precursor ion masses, for example, a difference of 14.0157 Da may correspond to methyl functionalizations, but in many cases the fragmentation spectra alone is not sufficient to establish with confidence the position of the modification and type of modification, and only a partial annotation (isomers) or a molecular class annotation can be proposed. A 14.0157 could be the result of methylation, or substitution of fatty acids (propionate vs butyrate) or amino acids (e.g. Gly vs Ala) due to catalytic promiscuity in the biosynthesis of the molecule. The use of *in silico* fragmentation can provide further insights on structure annotation when used to initiate the manual propagation starting from spectral library annotation [26]. It is important to mention that the connectivity of the network can change if we change the networking parameters, especially the parameters ‘Min Pairs Cos’ (the Minimum cosine score that must occur between a pair of consensus MS/MS) and the ‘Minimum Matched Fragment Ions’. This is one of the reasons for which in the ‘*Benchmarking NAP with a standard library*’ section we split the dataset in lower spectral similarity only (0.6 to 0.7 cosine score range) and reanalyzed it, having comparable result from the complete dataset (0.6 to 0.9 cosine score range). It is also worth mentioning that improvements on data acquisition as well as preprocessing can drastically optimize the networking, with for example, less redundant nodes and higher quality fragmentation spectra, and consequently optimize results from NAP [24, 49, 50].

The results show that NAP improves the rank of correct candidate structure generated by an *in silico* tool. The performance of NAP *Fusion* and *Consensus* scores were evaluated with two reference standard datasets (NIST library and CASMI 2016 challenges), and NAP’s use was highlighted with three experimental datasets (fecal samples, plant extracts and fungus garden samples). Overall, both NAP scores improve the rank of the correct structure from MetFrag. Moreover, when considering the compound class with ClassyFire, we observed that NAP improves the assignment of the correct compound structure in the first position from 29.0% with *Fusion* and 19.8% with *Consensus* to a better class assignment associated with the scoring to 38% with *Fusion* and 34% with *Consensus* (Fig 3), compared to *in silico* fragmentation performed alone at single spectrum level. With structure similarity clustering and class assignment one can begin to understand if there is an association between higher correct annotation rates for specific classes or structural motifs. This means that even when the actual structure may be incorrectly ranked, or absent from the database, the re-ranking annotated many structures with similar structural motifs instead. This is important as this would support a level 3 annotation and will allow end users to make informed decisions regarding the structural hypothesis of the molecules that could be detected by mass spectrometry. There is an association between lower annotation rates for specific classes or structural motifs, such as flavonoids, as there are often many isomeric structures possible for flavonoids and flavonoids do annotate within a structural family (Fig N in S1 Text and S10 Data).

One of the NAP’s challenges relies in the selection of meaningful structure databases. Previous studies have shown that large databases such as PubChem contain a high number of synthetic molecules that have distinct molecular features from molecules typically produced by living organisms [51]. For that reason, *in silico* fragmentation methods have used dedicated databases to search candidates [52], or used methods that improve natural product likeness in candidate ranking [10]. Therefore, we have used the largest naturally occurring small molecule databases, and offer the possibility for the user to select between popular databases (GNPS, HMDB, SUPER NATURAL, ChEBI) or to upload their own custom database. The PubChem library is also available to users, as an alternative for instances where no candidate structure is found in smaller targeted structural libraries, such as DNP, MarinLit or AntiBase for example [53]. We also provide additional code to guide users on database formatting from a list of InChI or SMILES (See Methods section).

We have used the combinatorial fragmentation approach (MetFrag) because of its generality and usability reported in recent studies [12, 54], but the network propagation method can be extended to other classes of *in silico* fragmentation approaches. We expect that the use of NAP has the potential to improve the performance of other *in silico* fragmentation annotation approaches [9–11, 55]. Tools such as CFM-ID can predict a fragmentation spectrum from a structure, and offer the flexibility to create *in silico* spectral libraries. However, the ability to retrain the classification model and to regenerate new *in silico* spectral libraries is essential to keep pace with the growing number of public spectral libraries. Additionally, further developments in NAP should integrate LC-MS processing tools in order to annotate the adduct type and predict the molecular formula with confidence. The integration of those tools will limit the search of candidates in structure databases to only those having the likely molecular formula(s) [56] and/or substructures [17], which will improve the ranking of the *in silico* tools, and the performance of NAP annotation. Additionally, alternative ways to propagate information or select neighbor candidates should be tested to improve propagation results [57].

The fecal, plant and fungal data sets revealed many expected molecules, the acetylated–saccharide family, the phorboid ester family and the sterols family, respectively. Sterol derivatives are common fungal metabolites, and have been isolated from medicinal fungi [58] and soil fungi [59–64]. The bioactivity range for this class of compounds include antibiosis [61] and anti-cancer, as it has been described for ergosterol peroxide [58]. Since sterols are fungal metabolites, their identification from this dataset is consistent. Now that the presence of ergosterol peroxide and oxygenated sterols has been observed in fungal gardens, and considering their potent biological activities [58, 61], their ecological role in the *T*. *septentrionalis* fungal gardens symbiotic system need to be elucidated.

We expect that network annotation propagations will increase with the deposit of new reference MS/MS spectra in searchable public spectral libraries and extensions of structural databases with potential candidate structures. *In silico* libraries have already been a part of annotations in Metlin, a metabolomics search engine, since 2005 [65], and more recently Metlin provides CFM-ID *in silico* predicted reference spectra as part of their search engine. LIPID MAPS [66] and LipidBlast [67] use predicted spectra for lipids and mzCloud uses *ab initio* predicted spectra. *In silico* propagated annotations are not yet a part of such search engines. When these become part of public reference libraries, it is critical that provenance of annotation is retained so that users can decide to rely on the annotation or not. Moreover, when associated to *in silico* fragmentation, annotation propagation has the potential to improve the structural hypothesis for many MS/MS annotations (both qualitative and quantitatively), especially if those are validated as *in silico* library entries by experts, and reused during future spectral library searches. To facilitate that process, NAP is integrated into the GNPS web-platform and the functionality allowing the user to add those expert curated putative annotations will become a part of GNPS public libraries, with provenance clearly indicated for the user. We anticipate that *in silico* network based propagation will be one key approach to fill in the dark matter of metabolomics annotations.

## Methods

### Experimental data generation

*Trachymyrmex septentrionalis* fungus garden samples were collected from across the Eastern USA. Authorization for collecting samples were previously obtained from the corresponding state department: State of New Jersey Department of Environmental Protection Division of Parks and Forestry State Park Service unnumbered Letter of Authorization; North Carolina Division of Parks and Recreation Scientific Research and Collecting Permit 2015_0030; Florida Department of Agriculture and Consumer Services unnumbered Letter of Authorization; Georgia Department of Natural Resources State Parks & Historic Sites Scientific Research and Collection Permit 032015; Department of Natural Resources Wildlife Resources Division unnumbered Letter of Authorization. Samples were extracted with 2:1 dichloromethane/methanol 3 times and dried under nitrogen. Samples were resuspended in 100% methanol containing 2µM sulfamethazine as internal standard and LC-MS/MS analysis was performed in an UltiMate 3000 UPLC system (Thermo Scientific) using a Kinetex 1.7 mm C18 reversed phase UHPLC column (50 X 2.1 mm) and Maxis Q-TOF mass spectrometer (Bruker Daltonics) equipped with ESI source. The column was equilibrated with 5% solvent B (LC-MS grade acetonitrile, 0.1% formic acid) for 1 min, followed by a linear gradient from 5% B to 100% B in 8 min, held at 100% B for 2 min. Then, 100%–5% B in 0.5 min and maintained at 5% B for 2.5 min at a flow rate of 0.5 mL/min throughout the run. MS spectra were acquired in positive ion mode in the range of 100–2000 m/z. A mixture of 10 mg/mL of each sulfamethazine, sulfamethizole, sulfachloropyridazine, sulfadimethoxine, amitriptyline, and coumarin-314 was run after every 24 injections for quality control. An external calibration with ESI-L Low Concentration Tuning Mix (Agilent technologies) was performed prior to data collection and internal calibrant Hexakis(1H,1H,3H-tertrafluoropropoxy)phosphazene was used throughout the runs. The capillary voltage of 4500 V, nebulizer gas pressure (nitrogen) of 2 bar, ion source temperature of 200°**C**, dry gas flow of 9 L/min source temperature. Spectral rate of 3 Hz for MS1 and 10 Hz for MS/MS, total cycle time range of 0.83 sec consisted of one full MS scan and up to 5 MS/MS scans; MS/MS active exclusion parameter was enabled, set to 2 and to release after 30 s, precursor ion was reconsidered for MS/MS if current intensity/previous intensity ratio >2; CID energies for MS/MS data acquisition were used as in Table 1:

**Table 1:** CID energies for MS/MS data acquisition.

Type	Mass	Width	Collision	Charge State
Base	100.00	4.00	22.00	1
Base	100.00	4.00	18.00	2
Base	300.00	5.00	27.00	1
Base	300.00	5.00	22.00	2
Base	500.00	6.00	35.00	1
Base	500.00	6.00	30.00	2
Base	1000.00	8.00	45.00	1
Base	1000.00	8.00	35.00	2
Base	2000.00	10.00	50.00	1
Base	2000.00	10.00	50.00	2

Basic stepping function was used to fragment ions at 50% and 125% of the CID calculated for each m/z from the above table with timing of 50% for each step. Similarly, basic stepping of collision RF of 550 and 800 Vpp with a timing of 50% for each step and transfer time stepping of 57 and 90 μs with a timing of 50% for each step was employed. The mass of internal calibrant was excluded from the MS/MS list using a mass range of m/z 921.5–923.5. The data were deposited in the online repository namely MassIVE (ftp://massive.ucsd.edu/MSV000081671).

### Structure database construction

Structures were downloaded from their respective databases GNPS (http://gnps.ucsd.edu/ProteoSAFe/gnpslibrary.jsp?library=all), HMDB (http://www.hmdb.ca/downloads), SUPER NATURAL (http://bioinf-applied.charite.de/supernatural_new/) and ChEBI (https://www.ebi.ac.uk/chebi/downloadsForward.do). The Dictionary of Natural Products (DNP) structures were downloaded manually using the institutional subscription. All structures were classified by ClassyFire [33] taxonomy classification using an *in house* script available at https://github.com/DorresteinLaboratory/NAP_ProteoSAFe/. An initial user manual is also available for NAP parameter setting, including ClassyFire classification based database candidates’ selection.

### Network calculation and data availability

The NIST17 library .msp file containing 574,826 spectra from various instruments, acquisition modes and adduct types was parsed with an *in house* script to recover all [M+H]^+^ unique compound spectra. From 11,331 spectra recovered, a subset of 5,467 NIST17 [M+H]^+^ unique compound presented at least one analog in the networking conditions described below and were retained for validation. The raw data was retrieved from the public MassIVE datasets for *Euphorbia dendroides* (ftp://massive.ucsd.edu/MSV000080502), fecal (ftp://massive.ucsd.edu/MSV000081120) and a fungal data set (ftp://massive.ucsd.edu/MSV000081671). The CASMI data was downloaded from (http://www.casmi-contest.org/2016/index.shtml). The networks were calculated using GNPS web interface, and can be accessed with the following job IDs: for NIST library - http://gnps.ucsd.edu/ProteoSAFe/status.jsp?task=aa3386fd782e4875b6109fb32a93eb5a, http://gnps.ucsd.edu/ProteoSAFe/status.jsp?task=229296bd89fc4eb19c4d4cb4e6d50744, http://gnps.ucsd.edu/ProteoSAFe/status.jsp?task=8ae2cbf410944c96a269e80670915851, http://gnps.ucsd.edu/ProteoSAFe/status.jsp?task=ee1233f263f94268ac62dc4cd358cd12, http://gnps.ucsd.edu/ProteoSAFe/status.jsp?task=00851eb8fb2c4050b581ab898b9d228e, http://gnps.ucsd.edu/ProteoSAFe/status.jsp?task=1f905afc070241a58c74672b40333ac0, http://gnps.ucsd.edu/ProteoSAFe/status.jsp?task=e6663cf00af64a928def7a70108a2b19, http://gnps.ucsd.edu/ProteoSAFe/status.jsp?task=f2f4d89b7dbc4fc0ae78591b36f71585, http://gnps.ucsd.edu/ProteoSAFe/status.jsp?task=c26168effc244aedb95fbea7de289aaf http://gnps.ucsd.edu/ProteoSAFe/status.jsp?task=7e45eb1bd3c34cc9a8a27bf20c182f4d; NIST library subnetwork with cosine score < 0.7 http://gnps.ucsd.edu/ProteoSAFe/status.jsp?task=8b0b5a467da9416b81ab4f925a4f4b43; for Fecal, *Euphorbia dendroides* extracts and Fungal dataset http://gnps.ucsd.edu/ProteoSAFe/status.jsp?task=f0cabc92247d44789900944a69874e8a, http://gnps.ucsd.edu/ProteoSAFe/status.jsp?task=ce2a564dbd704c0595494e04798b0233, http://gnps.ucsd.edu/ProteoSAFe/status.jsp?task=b753797b0dad4f1e84142dd59c84615b; and finally for CASMI negative mode http://gnps.ucsd.edu/ProteoSAFe/status.jsp?task=a3f02b1b648a43b6a210063a4ee2f787 and positive mode http://gnps.ucsd.edu/ProteoSAFe/status.jsp?task=231902c6d75f41df8403e454c96e8d4a. The parameters can be accessed by cloning the job or at the link “Networking Parameters and Written Network Description”. The corresponding NAP jobs can also be accessed through the web interface with the following job IDs: for NIST library - http://proteomics2.ucsd.edu/ProteoSAFe/status.jsp?task=29d517e67067476bae97a32f2d4977e0, http://proteomics2.ucsd.edu/ProteoSAFe/status.jsp?task=d270e79876cb48deb6aabd52a4fc647e, http://proteomics2.ucsd.edu/ProteoSAFe/status.jsp?task=e2125577fe2646129becc248b96d42ba, http://proteomics2.ucsd.edu/ProteoSAFe/status.jsp?task=81e01fe178d3424686079903d908b536, http://proteomics2.ucsd.edu/ProteoSAFe/status.jsp?task=daa546b038604e5f83eaafb811bd0313, http://proteomics2.ucsd.edu/ProteoSAFe/status.jsp?task=61c8a0d01309408f8ecceb5b31dab1a8, http://proteomics2.ucsd.edu/ProteoSAFe/status.jsp?task=60fe9f77b3d04789997bf19aa1a0a828, http://proteomics2.ucsd.edu/ProteoSAFe/status.jsp?task=53f8494ff9e8423697eebf4e98d287f0, http://proteomics2.ucsd.edu/ProteoSAFe/status.jsp?task=c93a840100ec49bdbb3c12e5ed1e4790, http://proteomics2.ucsd.edu/ProteoSAFe/status.jsp?task=5fd60b02f8ab4274bf45fd5b715b5e0b; NIST library subnetwork with cosine score < 0.7 http://proteomics2.ucsd.edu/ProteoSAFe/status.jsp?task=74e04164a8374929a4548655742c0a4f; for Fecal, *Euphorbia dendroides* extracts and fungal http://proteomics2.ucsd.edu/ProteoSAFe/status.jsp?task=9b6bddc2ba154b1397a53c7f7933430a, http://proteomics2.ucsd.edu/ProteoSAFe/status.jsp?task=8ae3aa45bfe449d7969975189b14f429, http://proteomics2.ucsd.edu/ProteoSAFe/status.jsp?task=fb02f64992bb4a0fb46b0e4832e69597; for CASMI positive http://proteomics2.ucsd.edu/ProteoSAFe/status.jsp?task=7c5a16eba2eb42f88647e7d21e57f1bc and for negative mode http://proteomics2.ucsd.edu/ProteoSAFe/status.jsp?task=e9745e159f6f433d9efb71e9813df29a. The parameters from NAP can be accessed by cloning the job through the web interface and S11 Data.

### Implementation and availability

The workflow was implemented using R and Python languages. The MetFrag 2.3 command line tool was downloaded from http://c-ruttkies.github.io/MetFrag/. The scoring *Fusion* used was $S_{c}=\alpha *f_{c}+(1-\alpha )\sum_{j=1}^{M}sig(m_{j}*t_{cj})$ previously described in MetFusion as: where *c* represents each MetFrag candidate, *f*_c_ the MetFrag score and the ‘spectral summary’ represents the spectral library search cosine scores *m*_j_ for all neighbor nodes *j*, the chemical similarity *t*_cj_ between MetFrag candidate *c* and each neighbor node result *j*. The *sig* represents the sigmoid function. We used as default the optimized parameters α = 0.3, β = -9 and γ = 0.6 [28]. The *Consensus* scoring uses the same scoring function, but instead of using the structural similarity of the spectral library search from the neighbor it uses the maximum structural similarity of up to *n first* candidates of each neighbor node.

The molecular fingerprints and structural similarity were calculated with the fingerprint R package (https://cran.rstudio.com/web/packages/fingerprint/index.html), using CDK version 1.5.13. We have used the ‘extended’ fingerprint type and tanimoto similarity for *Fusion* and *Consensus* scoring. The same fingerprint type and the dissimilarity calculated as 1—tanimoto similarity were used for clustering.

To group candidate structures we used the Dynamic Tree Cut method for dynamic branch cutting available in the dynamicTreeCut R package (https://labs.genetics.ucla.edu/horvath/CoexpressionNetwork/BranchCutting/). Before clustering we create a low dimensional (up to 10 dimensions) projection with Multidimensional Scaling of tanimoto dissimilarity matrices, and then cluster the new coordinates using hierarchical clustering with euclidean distance and ‘ward’ grouping method. After the creation of a dendrogram, the same is subjected to automated group detection (dynamicTreeCut) using the following parameters: minClusterSize = 2, method = "hybrid", deepSplit = 2.

The Workflow was implemented using ProteoSAFe (https://bix-lab.ucsd.edu/display/PS/XML+Configuration+Overview). The ProteoSAFe web interface can be found at http://proteomics2.ucsd.edu/ProteoSAFe/, under the workflow name NAP_CCMS and the code is available at github (https://github.com/DorresteinLaboratory/NAP_ProteoSAFe/).

### Evaluation

To create the evaluation, results of each analysis from NAP containing the original MetFrag, *Fusion* and *Consensus* scores were compared. Ranking ties among ranking methods were considered overlap between the methods. When ties were found inside candidate lists, all candidates were considered to have the same position (e. g. 1, 2, 2, 2, 3, … instead of 1, 2, 2, 2, 5, …). The random candidate assignment was determined by sampling from the list of candidates from each spectrum following a uniform distribution.

## Supporting information

S1 Text Supplementary Figures for model validation and manual inspection. (DOC)

S1 Data Assessment of *n first* parameter impact on *Consensus* scoring. (XLS)

S2 Data Mean and median ranking position for MetFrag and subsequent *Consensus* re-ranking of candidate structures. (XLS)

S3 Data General overview of NIST benchmarking dataset annotation results. (XLS)

S4 Data Mean, median and first position ranking position for MetFrag *Fusion* and *Consensus* re-ranking of candidate structures. (XLS)

S5 Data Detailed information of NIST benchmark selection of spectra with cosine score < 0.7 in the network. (XLS)

S6 Data Detailed information of all NIST benchmark selection. (XLS)

S7 Data Detailed information of evaluated CASMI 2016 positive mode data. (XLS)

S8 Data Detailed information of evaluated CASMI 2016 negative mode data. (XLS)

S9 Data Manually inspected clustered spectra annotation for fungal garden dataset. (XLS)

S10 Data Class error association test for NIST benchmark dataset. (XLS)

S11 Data NAP parameters for each dataset described in the manuscript. (XLS)
