# Supplementary material for: Propagating annotations of molecular networks using in silico fragmentation
Source: PLoS Comput Biol. 2018 Apr 18;14(4):e1006089. doi: 10.1371/journal.pcbi.1006089 (PMC5927460; doi:10.1371/journal.pcbi.1006089)
Supplement: S1 Text — (DOC) [file pcbi.1006089.s001.doc]

## **Supplementary Material**

## **Propagating annotations on molecular networks using *in silico* fragmentation**

Ricardo R. da Silva**^1,2^,** Mingxun Wang^1^, Louis-Félix Nothias^1^, Justin J. J. van der Hooft^1,3^, Andrés Mauricio Caraballo-Rodríguez^1^, Evan Fox^4^, Marcy J. Balunas^5^, Jonathan L. Klassen^4^, Norberto Peporine Lopes^2^, Pieter C. Dorrestein^1,*^

^1^Collaborative Mass Spectrometry Innovation Center, Skaggs School of Pharmacy and Pharmaceutical Sciences, University of California, San Diego, La Jolla, CA, USA

^2^NPPNS, Department of Physics and Chemistry, School of Pharmaceutical Sciences of Ribeirão Preto, University of São Paulo, Ribeirão Preto, SP, Brazil

^3^Bioinformatics Group, Department of Plant Sciences, Wageningen University, Wageningen, The Netherlands.

^4^Department of Molecular and Cell Biology, University of Connecticut, Storrs, CT, USA

^5^Division of Medicinal Chemistry, Department of Pharmaceutical Sciences, University of Connecticut, Storrs, CT, USA


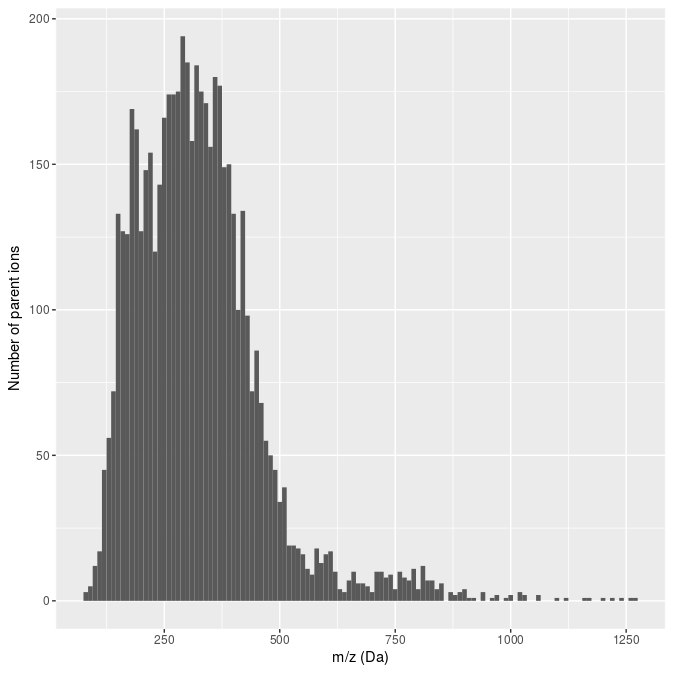


Fig A – Distribution of 5,467 [M+H]^+^ parent ions (m/z) from NIST17 subset spectra.


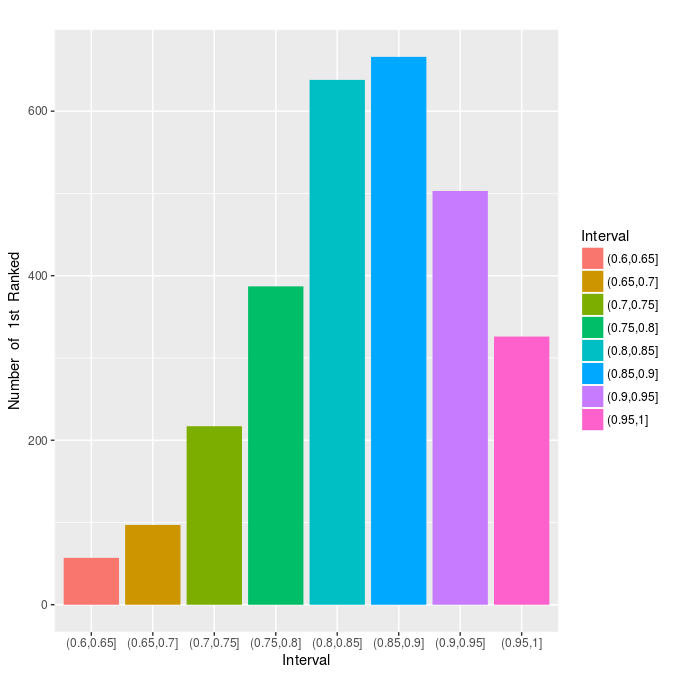


Fig B – Total number of query [M+H]^+^ spectra correctly annotated by NAP (*Fusion* scoring) for each interval of average cosine score to its direct neighbor.


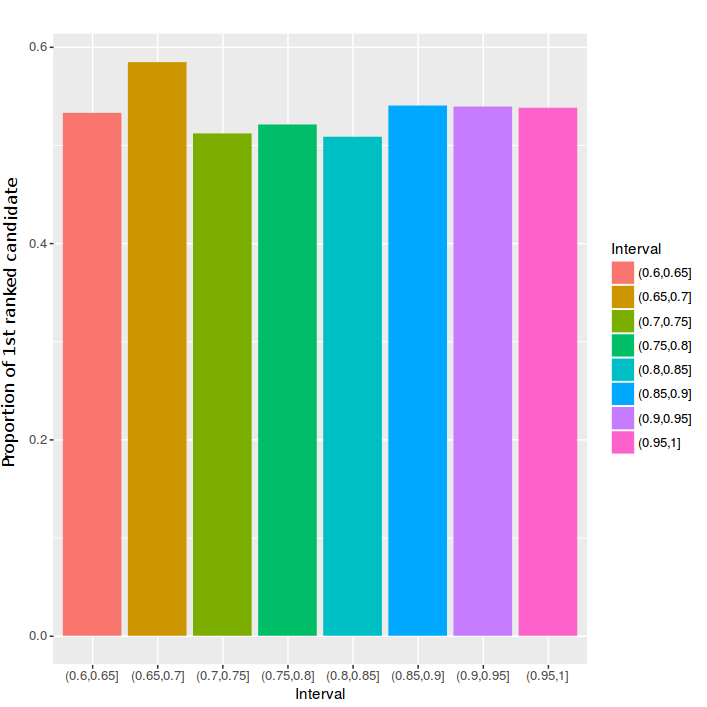


Fig C – Proportion of query [M+H]^+^ spectra correctly annotated by NAP (*Fusion* scoring) for each interval of average cosine score to its direct neighbor.


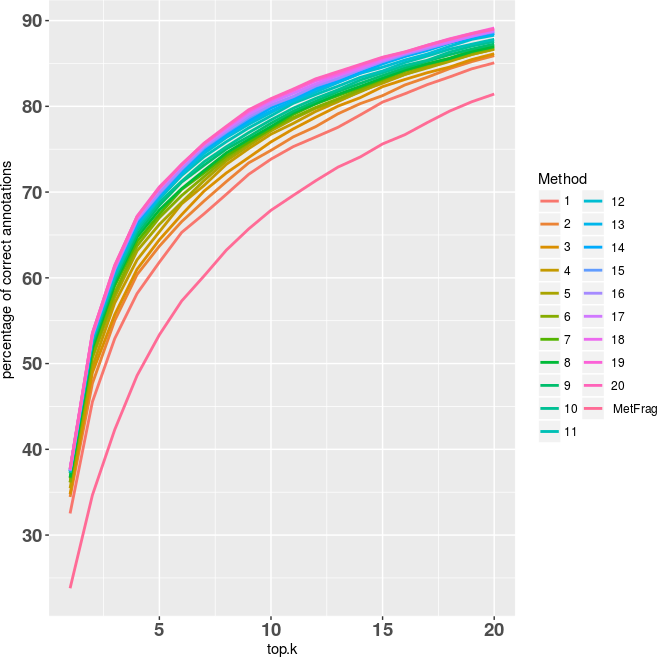


Fig D - NAP re-ranking assessment using the 5,467 NIST17 [M+H]^+^ benchmark dataset that have known structures for clustered spectra in the molecular network. The impact of percentage of correct annotations as influenced by setting the *n-first* (1 to 20) parameter change for network *Consensus* scoring is illustrated.


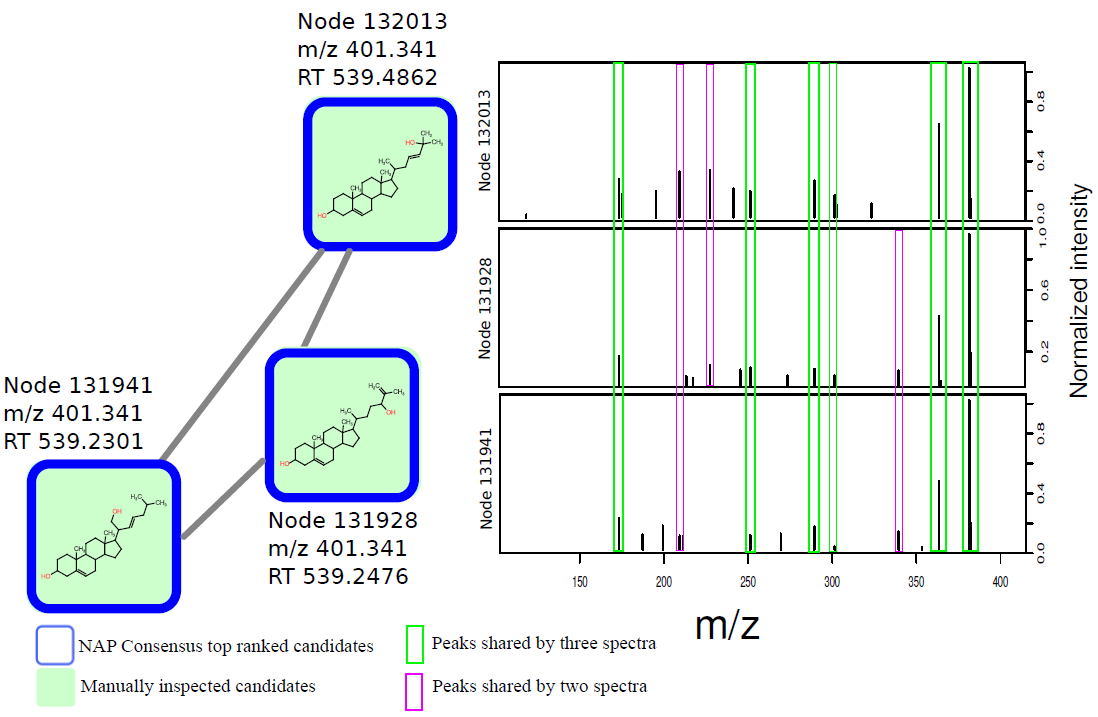


Fig E – Annotation propagation behavior illustration on nodes potentially incorrectly clustered during the networking process (See Figure 1 in the main text). The three nodes are an excerpt of Figure 6 c) in the main text, which shows how *Consensus* scoring was able to retrieve structures with the same structural backbone, in agreement with spectral library annotation, improving MetFrag candidate ranking. The nodes in the network represent a scenario in with the same parent mass and a very close retention time are observed, and for which different positional isomers were attributed as first ranked candidate. The most intense fragment peaks are also the same among the spectra.

Fig F – Overlap between ranking improvements for correct structure assigned to query spectra by *Consensus* scoring using different *n-first* parameters (from 1 to 20).


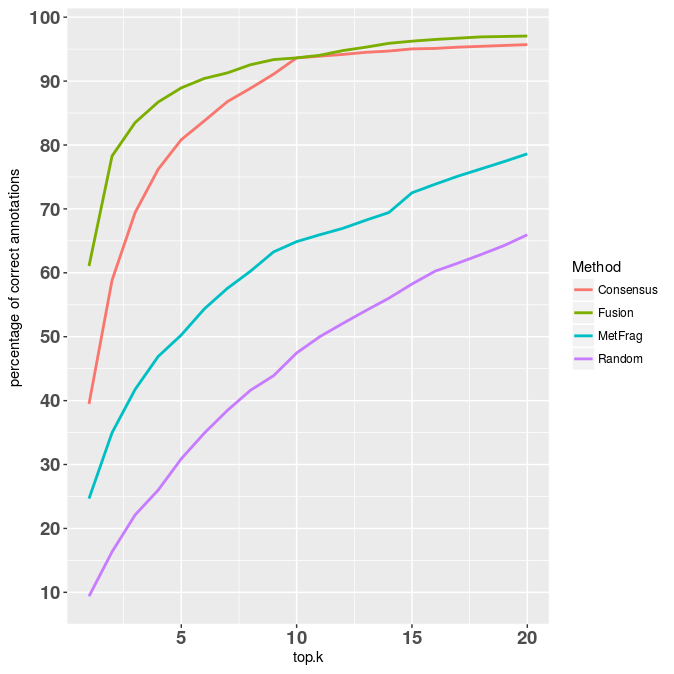
Fig G - NAP re-ranking assessment using the 1,734 NIST17 [M+H]^+^ benchmark data sub set consisting of a network with only edges having a cosine score < 0.7.


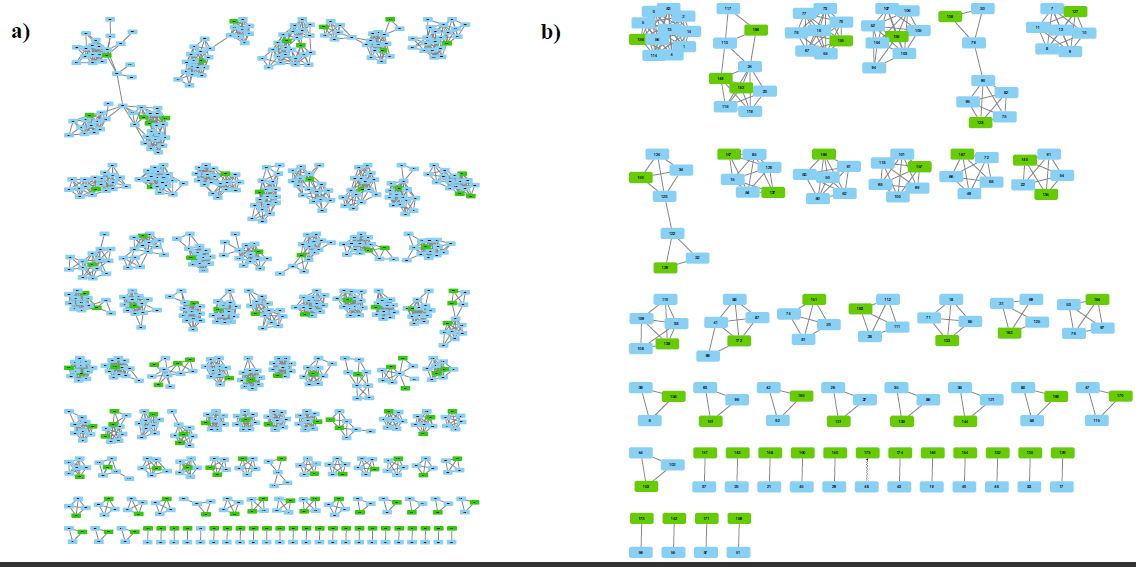


Fig H – Spectral network from CASMI 2016 and GNPS public libraries spectra, showing in green the CASMI spectra. a) Positive mode. b) Negative mode. The network only contains connected components with two or more nodes for which one or more spectra from CASMI 2016 had an analog in spectral libraries.


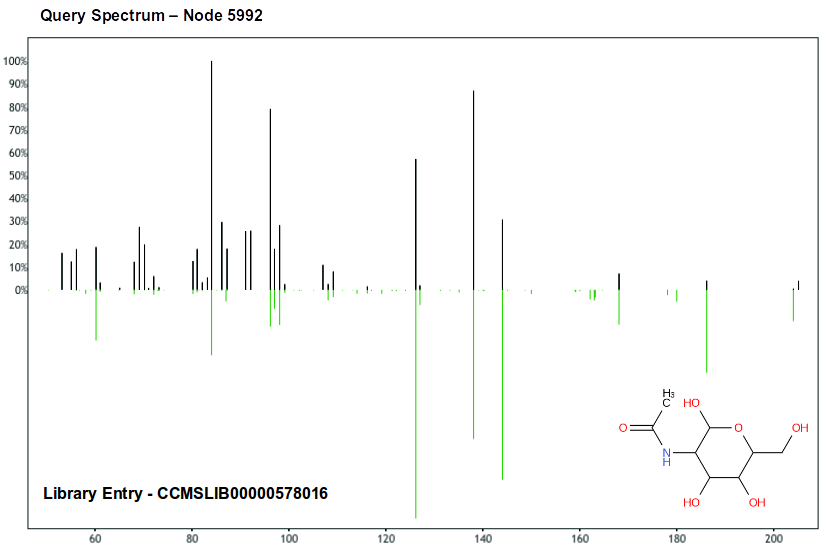


Fig I – Spectral library match from Fecal data set network (<http://gnps.ucsd.edu/ProteoSAFe/status.jsp?task=f0cabc92247d44789900944a69874e8a>), showing the query spectrum, black on top and the GNPS filtered library entry, green on the bottom.


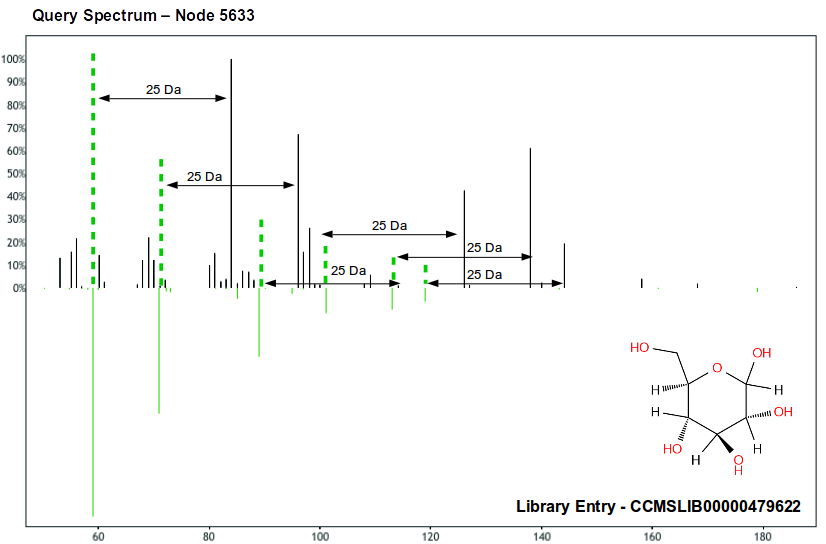


Fig J – Spectral library match from Fecal data set network (<http://gnps.ucsd.edu/ProteoSAFe/status.jsp?task=f0cabc92247d44789900944a69874e8a>), showing the query spectrum, black on top and the library entry, green on the bottom. Arrows show the mass shift between the query parent mass (204.09) and library entry parent mass (179.063). We believe this is an incorrect match based on manual inspection.

Fig L - a) Top 7 matched hits from MzCloud database to MS/MS spectrum of precursor mass 294.118, a neighboring node of N-acetylglucosamine/N-acetylgalactosamine. b) MzCloud mass fragment structural annotations of N-acetylglucosamine.

Fig M - NAP annotation with top scoring matches using NAP network *Consensus* scoring (without previous ranking with *Fusion*), showing candidate lists associated to incorrectly annotated nodes with the correct candidate highlighted in blue.


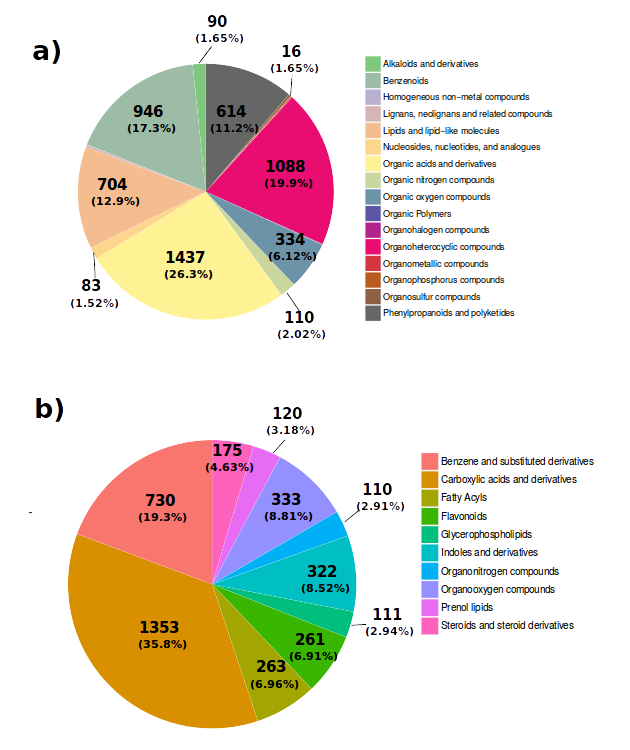


Fig N - Distribution of ClassyFire chemical classes for the 5,467 NIST17 [M+H]+ spectra. a) Super class. b) Top 10 most abundant Classes.
